# Evaluation of Occurrence, Concentration, and Removal of Pathogenic Parasites and Fecal Coliforms in Three Waste Stabilization Pond Systems in Tanzania

**DOI:** 10.1155/2019/3415617

**Published:** 2019-10-23

**Authors:** Abdallah Zacharia, Wajihu Ahmada, Anne H. Outwater, Billy Ngasala, Rob Van Deun

**Affiliations:** ^1^Department of Parasitology and Medical Entomology, Muhimbili University of Health and Allied Sciences, Dar es Salaam, Tanzania; ^2^Department of Chemical and Mining Engineering, University of Dar es Salaam, Dar es Salaam, Tanzania; ^3^Department of Community Health Nursing, Muhimbili University of Health and Allied Sciences, Dar es Salaam, Tanzania; ^4^Unit Life Sciences and Chemistry, Thomas More University of Applied Sciences, Geel, Belgium

## Abstract

In Tanzania, waste stabilization ponds (WSPs) are employed to treat wastewater, and effluents are used for urban agricultural activities. The use of untreated or partially treated wastewater poses risks of disease transmission, including parasitic and bacterial infections, to exposed communities. Little is known about the occurrence, concentration, and removal of parasites and fecal coliform (FC) bacteria in WSPs in Tanzania. This study evaluates the occurrence and concentration of parasites and FCs in wastewater, the efficiency of WSPs in removing parasites and FCs, and the validity of using FCs as an indicator of parasites. This was a cross-sectional study conducted between February and August 2018. Wastewater samples were collected from three WSPs located in the Morogoro, Mwanza, and Iringa regions. APHA methods were used to test physicochemical parameters. The modified Bailenger method and Ziehl–Neelsen stain were used to analyse parasites. Membrane filtration method was used to analyse FCs. Data were analysed using IBM SPSS version 20. Helminth egg removal ranged from 80.8% to 100%. Protozoan (oo)cyst removal ranged from 98.8% to 99.9%. The Mwanza WSP showed the highest FC reduction (3.8 log units (100 mL)^−1^). Both the parasites and FCs detected in the effluents of assessed WSPs were of higher concentrations than World Health Organization and Tanzania Bureau of Standards limits, except for helminths in the Morogoro WSP and FCs in the Mwanza WSP. FCs were significantly correlated with protozoa (*p* < 0.01) and predicted protozoa occurrence well (*p*=0.011). There were correlations between physicochemical parameters, parasites, and FC bacteria in the WSP systems. Inadequate performance of these systems may be due to lack of regular maintenance and/or systems operating beyond their capacity. FC indicators were observed to be a good alternative for protozoa monitoring, but not for helminths. Therefore, during wastewater quality monitoring, helminths should be surveyed independently.

## 1. Introduction

In Tanzania, waste stabilization ponds (WSPs) have been used for municipal wastewater treatment for several decades [1]. Effluents from various WSP systems are used for urban agricultural activities, such as vegetable gardening in Iringa [2], and vegetable and rice paddy farming in Moshi [3] and Morogoro [4]. Using WSP effluent for irrigation was observed to benefit farmers. For example, in a study conducted in Morogoro, field plots irrigated with effluents and without artificial fertilizers were reported to produce more vegetables and rice than tap water irrigated plots, with and without artificial fertilizers [5]. Moreover, farmers using WSP effluents in Moshi were reported to grow paddy twice a year, as compared to farmers practicing rainwater agriculture [3].

Despite the abovementioned advantages, the use of untreated or partially treated wastewater poses a risk of disease transmission to farmers and the community [6]. Apart from the transmission of pathogenic bacteria, another public health concern about the use of wastewater is the transmission of parasitic diseases. Wastewater-associated pathogenic parasites are considered pathogens of great public health importance due principally to their environmentally persistent transmissive stages, a low infective dose (1–10 eggs for helminths, and 10–100 (oo)cysts for protozoa per person) [7, 8], limited or transient acquired immunity, and morbidity, particularly in immunocompromised hosts [9].

It has been hypothesized that lack of operation and maintenance of wastewater treatment systems, such as WSPs, decreases the removal of pathogens (parasites) and fecal indicators, thus creating uncertainty about the risks associated with wastewater use [10]. In Tanzania, many WSP systems were reported to be dilapidated and receive inadequate care [11]. Moreover, there is a paucity of information about the occurrence of parasites in wastewater and the efficiency of WSPs in removing parasites from wastewater. Few studies have evaluated these systems based on the removal of indicator bacteria. In addition, the National Environmental Standards Compendium of the Tanzania Bureau of Standards (TBS) specifies coliform bacteria as an indicator of wastewater pathogens, including wastewater-transmitted pathogenic parasites [12]. However, recent studies have shown variation in indicator bacteria correlation with parasites in wastewater, questioning its use in predicting the occurrence and removal of parasites from wastewater [13, 14].

The aims of this study were to (1) determine the occurrence and concentrations of wastewater-related pathogenic parasites and fecal coliform (FC) indicator bacteria in three selected Tanzanian WSPs and (2) evaluate their performance in removing pathogenic parasites and FC indicator bacteria. The correlation between physicochemical parameters, FC indicator bacteria, and pathogenic parasites (helminths and protozoa) was also examined.

## 2. Materials and Methods

### 2.1. Study Design

This was a cross-sectional study conducted between February and August 2018. Three WSP treatment systems located in the Morogoro, Mwanza, and Iringa regions in Tanzania were evaluated.

### 2.2. Study Sites

#### 2.2.1. Mwanza WSPs

**Figure 1:**
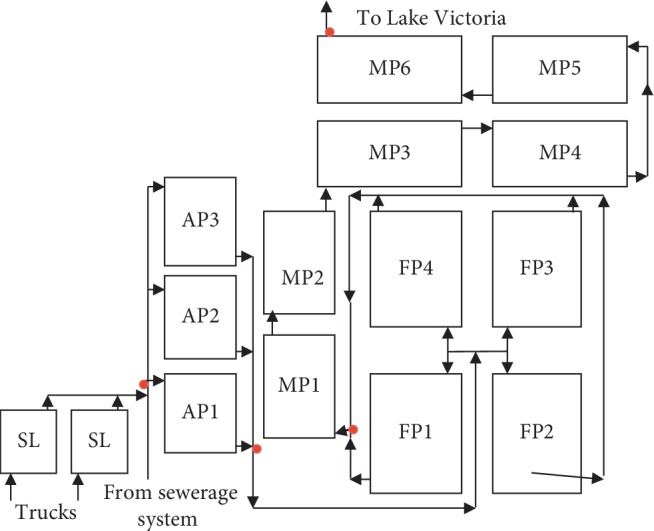
Schematic presentation of Mwanza waste stabilization ponds. Note: SL = septage lagoon; AP = anaerobic pond; FP = facultative pond; MP = maturation pond. Red dots indicate sampling points.

The site in Mwanza is located in Butuja sub-ward, at 2°28′09.37″S 32°54′35.49″E. The system is owned by the Mwanza Urban Water Supply and Sanitation Authority (MWAUWASA) and serves about 3,500 connected households. The design of this system consists of 2 septage lagoons receiving fecal sludge delivered by vacuum trucks, 3 anaerobic ponds connected in parallel, 4 facultative ponds in parallel, and 6 maturation ponds in a series (Figure 1). This treatment system has the capacity of receiving 5,000 m^3^·d^−1^ of wastewater. Effluent from this system is discharged into Lake Victoria.

#### 2.2.2. Morogoro WSPs

**Figure 2:**
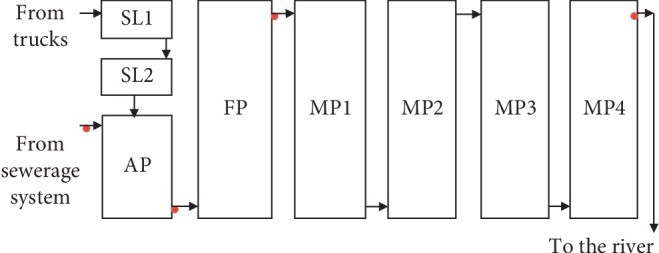
Schematic presentation of Morogoro waste stabilization ponds. Note: SL = septage lagoon; AP = anaerobic pond; FP = facultative pond; MP = maturation pond. Red dots indicate sampling points.

WSPs in Morogoro are owned and operated by the Morogoro Urban Water Supply and Sanitation Authority (MORUWASA) and are located in Mafisa, Morogoro (at 6°47′07.28″S 37°40′29.34″E). The treatment system consists of 2 septage lagoons, 1 anaerobic pond, 1 facultative pond, and 4 maturation ponds connected in a series (Figure 2). The effluent from the treatment system is drained into a small river which supports a wetland with about 8 hectares of rice fields and is used further downstream for vegetable irrigation.

#### 2.2.3. Iringa WSPs

**Figure 3:**
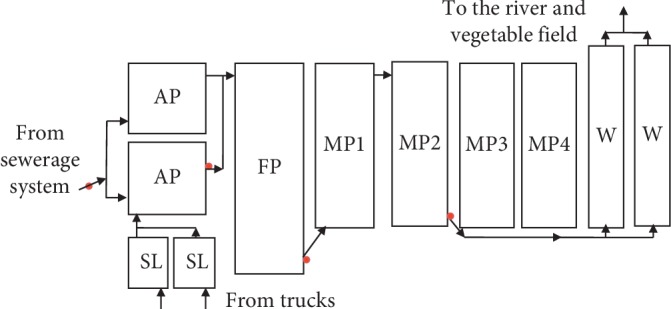
Schematic presentation of Iringa Municipal waste stabilization ponds. Note: SL = septage lagoon; AP  = anaerobic pond; FP = facultative pond; MP = maturation pond; W = constructed wetland. MP3 and MP4 are newly constructed maturation ponds, which were not in operation during the study period. Red dots indicate sampling points.

The Iringa wastewater treatment plant is located at 7°45′31.47″S 35°40′12.34″E in Don Bosco. The treatment system is owned by Iringa Urban Water Supply and Sanitation Authority (IRUWASA). The system has 2 septage lagoons receiving fecal sludge delivered by vacuum trucks, 2 anaerobic ponds operating in parallel, 1 facultative pond, and 2 maturation ponds connected in a series (Figure 3). The effluent is discharged to free surface wetlands and then to a stream near Hoho Street and used by local residents for irrigation and brickmaking.

### 2.3. Wastewater Sample Collection, Storage, and Transportation

Wastewater samples were collected for analysis of physicochemical (1 L), parasitological (10 L, which was allowed to settle for 2 h and 1 L sediment was taken), and bacteriological (250 ml) parameters at intervals of one month for 4 or 5 visits. During each visit to Morogoro, Mwanza, and Iringa WSPs, a total of 4 samples were collected, one from each sampling point, as indicated in Figures 1–3.

Wastewater samples for chemical oxygen demand (COD) and total nitrogen (TN) tests were preserved by using sulphuric acid at pH of 2; those for parasitological analysis were preserved by using 10% formaldehyde. All samples were transported to the respective laboratory in a cool ice box (at a temperature of 4°C) within 6–36 h after collection. The samples for physicochemical and bacterial tests were transported to the University of Dar es Salaam water resources laboratory, and those for parasitological tests were transported to the Muhimbili University of Health and Allied Sciences parasitology laboratory. All samples were analysed immediately upon arrival at the laboratory.

On-site measurement of wastewater flow rates, temperature, and pH was conducted during sample collection. Wastewater flow rates were measured using a stopwatch and tracer method to detect the velocity of wastewater within a channel. The velocities of wastewater were multiplied to the channel cross-sectional area. The procedure was repeated thrice and the average was computed. Hydraulic retention time (HRT) of each system during the study period was estimated based on the calculated flow rate and designed volume of the system. Wastewater temperature and pH were determined by using a pH meter (Sartorius, Model: PT-15) by directly inserting the probe into the wastewater.

### 2.4. Wastewater Analysis for Physicochemical Parameters

In order to determine the performance of WSP operations, physicochemical parameters were measured following “Standard methods for the examination of water and wastewater” [15]. These parameters included COD tested using the closed reflux colorimetric method (method 5220D), total suspended solids (TSS) using method 2540D, and TN using method 4500-N C.

### 2.5. Wastewater Analysis for Microbiological Parameters

Parasites were analysed by using the modified Bailenger method (MBM) as described in “Analysis of wastewater for use in agriculture—A laboratory manual of parasitological and bacteriological techniques” by Ayres and Mara [16]. Since *Cryptosporidium* oocysts are very small, they can be difficult to detect in the MBM, so the smears were stained to increase visualization, in accordance with the modified Ziehl–Neelsen (ZN) procedure [17]. Quantification of parasites was done using the following equation:

(1) $$\begin{array}{c} n=\frac{ax}{pv}, \end{array}$$

where *n* = number of eggs or (oo)cysts L^−1^ of wastewater, *a* = number of eggs or (oo)cysts counted, *x* = volume of the final product (mL), *p* = volume examined (0.15 mL for MBM and 0.05 ml for ZN), and *v*=original sample volume (L).

Membrane filtration technique was used to detect and quantify FC bacteria in wastewater samples [16]. Membrane filters with 0.45 *μ*m pore size and 47 mm diameter and m-Fecal broth selective media were used. The FC bacteria results were reported in colony-forming units (cfu) (100 ml)^−1^ of wastewater.

### 2.6. Data Analysis

Statistical analysis was performed using statistical software IBM SPSS version 20. Percentage reductions of physicochemical parameters and parasites by the system or a particular stage of the system were calculated by using the following equation:

(2) $$\begin{array}{c} p=\frac{\left(µ\text{i}-µ\text{e}\right)\times 100}{µ\text{i}}, \end{array}$$

where *p* = percentage reduction, *μ*i = mean concentration of the influent samples, and *μ*e = mean concentration of effluent samples.

For statistical analysis and log reduction computation, FC data were transformed to log units. Log reduction of FCs was calculated by substituting the average influent log concentration by the average effluent log concentration. Shapiro–Wilk tests were used to test for normality of the data. Data were considered normally distributed at *p* > 0.05. Kruskal–Wallis test was conducted to compare medians of parasites (protozoa and helminths) and FC concentrations between the influent and effluent samples of the three treatment systems. The nonparametric Spearman rank-order correlation was conducted to determine if there were any relationships between physicochemical parameters and FC, protozoan, and helminth concentrations in the WSP wastewater samples. A simple binary logistic regression model was performed to assess the ability of the FC indicator to predict the occurrence of helminths and protozoan parasites in WSP systems.

## 3. Results and Discussion

A total of 112 wastewater samples were collected from the three treatment systems and analysed for the presence and concentration of pathogenic parasites and FC bacteria. Twelve wastewater samples, four from each treatment plant, were analysed for physicochemical parameters.

### 3.1. Physicochemical Characteristics of Influent and Effluent Wastewater

The average wastewater flow rates were 6264, 5952, and 4416 m^3^·d^−1^, leading to theoretical HRT of 9, 12, and 5 d in Morogoro, Mwanza, and Iringa WSPs, respectively. The concentrations of COD, TSS, and TN vary between the influent and effluent samples of the three WSP treatment systems. The concentrations were higher in the influent samples than the effluent samples. The concentration of COD from the three WSP systems ranged from 420 to 815 mg/L in the influents and from 200 to 235 mg/L in the effluents. The concentration of TSS in the influents from the three WSP systems ranged from 135 to 494 mg/L and from 57 to 154 mg/L in the effluents. The concentration of TN in the influents from the three WSP systems ranged from 39 to 65 mg/L and from 32 to 52 mg/L in the effluents. The overall COD, TSS, and TN removal efficiency ranged from 52.4% in Morogoro to 71.2% in Iringa WSPs; 58% in Morogoro to 69% in Iringa WSPs; and 19% in Morogoro and Iringa WSPs to 33% in Mwanza WSPs (Table 1). Despite having a short theoretical HRT and a higher concentration of TSS in the influents, Iringa WSPs achieved the highest percentage reduction of both COD and TSS compared to the other two systems. Lower reduction of COD and TSS in the other two systems could be due to algae growth, decreased HRT due to sludge accumulation, and systems operating beyond their designed capacity. The TBS requires that wastewater effluent quality discharge into the environment has less than 60 mg/L COD, and 100 mg/L TSS [12]. The three WSP systems produced effluents with concentrations of COD that exceeded the set quality standard. Similar results for COD effluent concentrations were also reported in other WSPs in Tanzania [3, 18]. The WSPs in Morogoro achieved the required TSS effluent quality, while those of Mwanza and Iringa had slightly higher concentrations.

There were slight variations in pH and temperature between the influent and effluent samples of the three WSPs; the exception was the influent temperatures of Iringa WSPs, which were much lower than those of the other systems (Table 1). Within the systems, pH was observed to be higher in the effluent samples than the influent samples. The pH ranged from 7.0 to 7.5 for the influents and 8.1 to 8.3 for the effluents. The increase in pH in WSPs may be attributed to photosynthesis by pond algae, which consume more CO_2_ than can be restored by bacterial respiration, resulting in carbonate and bicarbonate dissociation. The resulting hydroxyl group from carbonate and bicarbonate dissociation accumulated in the water, leading to raised pH [18, 19]. The effluent pH qualities were within the TBS limit of 6.5 to 8.5 required for safely discharging wastewater into the environment [12]. Except for the Morogoro WSPs, wastewater temperatures were observed to increase as they passed through the ponds. Similar results were reported in Lugalo WSPs by Kaseva et al. [18], and it could be due to the effect of solar heating. The temperature of the effluents met the TBS discharge limit of 20–35°C [12].

### 3.2. Parasite Occurrence in Wastewater of the Three Treatment Systems

**Figure 4:**
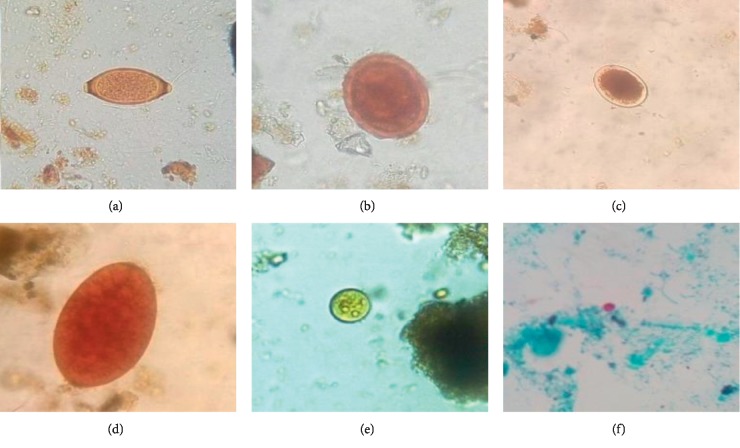
Images of some identified parasites in wastewater samples collected from either of the three WSP systems: (a) *Trichuris trichiura* egg, (b) *Ascaris lumbricoides* egg, (c) hookworm egg, (d) *Fasciola* spp egg, (e) *Entamoeba coli* cyst, and (f) *Cryptosporidium* spp oocyst (photos by first author).

Figure 4 presents photographs of some eggs and (oo)cysts of identified parasite species found in the wastewater of the three WSP systems. We identified a total of 11 parasite species. The helminthic parasites were nematodes: *Ascaris lumbricoides*, hookworm, *Trichuris trichiura* and *Trichostrongylus* spp, trematodes: *Fasciola hepatica*, and cestodes: *Taenia* spp. The protozoan species included flagellates: *Giardia lamblia*, amoebae: *Entamoeba histolytica* and *Entamoeba coli*, and coccidia: *Cryptosporidium* spp and *Isospora* spp. All these parasite species except for *Isospora* spp have been identified in wastewater in other African countries, including Tanzania's neighbouring countries Kenya and Uganda [20].

Forty out of 56 analysed wastewater samples (71%) were positive for at least one parasite. The percentage of positive samples was higher than the results obtained in other studies conducted in Africa. In Burkina Faso, Kpoda et al. [21] revealed 36% of samples were positive while assessing helminth and protozoa removal by WSP systems. All 14 (100%) of our raw/influent wastewater samples were positive for parasites. Similar findings were obtained by Grimason et al. [22] in Meze, France, whereby *Giardia* spp cysts were isolated from all 26 raw wastewater samples; by Stott et al. [23] in Ismailia, Egypt, whereby helminth eggs were detected in all raw wastewater samples; and by Konaté et al. [24] in Ouagadougou, Burkina Faso, whereby helminth eggs and/or protozoan cysts were isolated from all raw wastewater samples.

The occurrence of parasites in effluent samples of the three treatment systems was 40%, 80%, and 50% in Morogoro, Mwanza, and Iringa WSPs, respectively. The high percentage of parasite occurrence in Mwanza WSP effluent was contributed by a higher occurrence of *Trichostrongylus* spp eggs (60%) than in influent samples (20%). A similar case was reported by Ellis et al. [25] in Cayman Island WSPs, whereby hookworm eggs were isolated more frequently in effluents than influents. The higher occurrence may be due to the fact that *Trichostrongylus* spp parasite is multi-host and can infect a wide range of organisms [26]; hence, there is a possibility of on-site contamination by other infected animals in contact with wastewater in the systems. According to Ellis et al. [25], the reasons for more frequent detection of parasite eggs in effluents than influents could be due to the effect of thermal stratification in the final maturation pond, wind mixing of the pond content, and/or temperatures above 30°C (the case of Mwanza WSPs), leading to buoyancy of parasites by produced gas bubbles.

Hookworm eggs and *Entamoeba coli* cysts were detected in 21% and 61% of all samples, and were the most frequently recovered helminth and protozoan parasites, respectively. The results are supported by epidemiological studies conducted in several areas of the country, which show that hookworm eggs and *Entamoeba coli* cysts have higher prevalences than other helminths and protozoan parasites, respectively [27, 28]. The least frequently identified parasites were protozoa: *Isospora* spp, and helminths: *Taenia* spp and *Fasciola hepatica*.

### 3.3. Parasite Mean Concentration and Removal Efficiencies

In raw/untreated wastewater, the mean helminth concentrations were 12, 67, and 49.5 eggs L^−1^ in Morogoro, Mwanza, and Iringa WSPs, respectively. The helminth concentrations were lower than those detected in other low- and middle-income countries (70–3000 eggs L^−1^) and higher than those recovered in high-income countries (1–9 eggs L^−1^) [29]. The mean protozoan concentrations were 358, 231, and 669.5 (oo)cysts L^−1^ in Morogoro, Mwanza, and Iringa WSPs, respectively. Protozoan concentrations were comparable to those detected in other African countries, such as reported in Tunisia (250–590 cysts L^−1^) [30], in Nigeria (190 cysts L^−1^) [31], and in Burkina Faso (147 and 111 cysts/L) [32].

The combined data of raw/untreated wastewater samples from all treatment systems revealed the overall helminth mean concentration of 42.9 eggs L^−1^ and protozoan mean concentration of 422.3 (oo)cysts L^−1^. The contribution of each helminth species in the overall concentrations was hookworm 32.2%, *Ascaris lumbricoides* 31.9%, *Trichuris trichiura* 15.6%, *Trichostrongylus* spp 14.0%, and *Fasciola* spp 6.3%. Protozoan parasites were *Entamoeba coli* 76%, *Entamoeba histolytica*/*dispar* 14%, *Cryptosporidium* spp 7.8%, *Giardia* spp 2%, and *Isospora* spp 0.2%.

*Entamoeba coli* cysts were more frequently identified at higher concentrations than all other parasite species detected in all analysed wastewater samples. This protozoan parasite is a nonpathogenic species of *Entamoeba* that frequently exists as a commensal parasite in the human gastrointestinal tract. This parasite was reported because *Entamoeba coli* can be confused during microscopic diagnosis with the pathogenic *Entamoeba histolytica*. Moreover, when this parasite is detected in domestic wastewater, it is an indication of unhygienic conditions present in the sewered community, whereby people have consumed fecally contaminated products with the possibility of pathogenic organisms being consumed at the same time [33].

Specific concentrations of parasite species in wastewater may be attributed to their prevalence in the served communities. Parasite species with higher concentrations in wastewater were observed to have a higher prevalence in epidemiological studies. For example, Mazigo et al. [27] in the Mwanza region reported the following prevalences of parasites among hospitalized patients: hookworm 25.2%, *Ascaris lumbricoides* 1.6% and *Trichuris trichiura* 0.79% for helminths, and *Entamoeba histolytica/dispar* 13.6% and *Giardia lamblia* 6.9% for protozoa. Another study conducted by Siza et al. [34] to determine the prevalence of soil-transmitted helminths among school children in the four regions of Lake Zone showed the following results: hookworm 14.6%, *Ascaris lumbricoides* 3.2%, and *Trichuris trichiura* 0.3%. Moreover, a study by Venkatajothi [28] in the Dar es Salaam region to determine the incidence of intestinal protozoa among school children showed higher prevalence of *Entamoeba coli* (56% boys, 66% girls), followed by *Entamoeba histolytica* (31% boys, 26% girls) and *Giardia lamblia* (13% boys and 8% girls).

**Figure 5:**
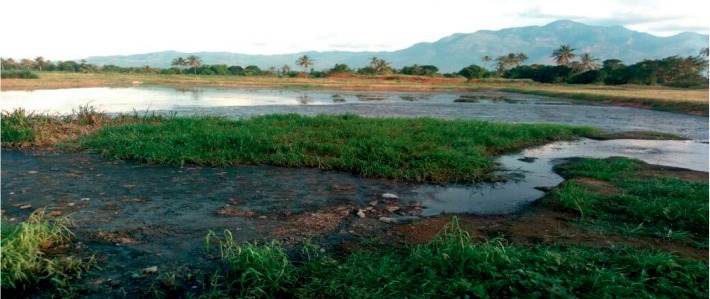
Anaerobic pond of Morogoro WSPs showing accumulated sludge (photos taken by first author during sample collection).

Parasite removal at each treatment step of the Morogoro, Mwanza, and Iringa WSPs is presented in Tables 2–4, respectively. In all treatment systems, higher concentrations of parasites were removed in anaerobic ponds, except for protozoa in Morogoro WSPs. The anaerobic pond in Morogoro WSPs was observed to have accumulated sludge that extended above the surface of the pond as islands (Figure 5). The sludge accumulation may have contributed to the inadequate removal of protozoa. The excessive accumulation of sludge affects pond hydraulics, creating short-circuiting that may carry parasite eggs, cysts, or oocysts through to the outlet, or resuspend eggs and (oo)cysts that have been deposited in the pond sediments [10]. Helminths were reduced by 93.3%, 96.7%, and 75.8% in the anaerobic ponds of Morogoro, Mwanza, and Iringa, respectively. Protozoa decreased from 368 to 303.2, 231 to 12, and 669.5 to 55.3 (oo)cysts L^−1^ in the anaerobic ponds of Morogoro, Mwanza, and Iringa WSPs, respectively. The higher performance of anaerobic ponds for removal of parasites was also reported by Konaté et al. [24] in Burkina Faso. However, these ponds are prone to sludge accumulation as they receive raw wastewater, thus reducing their efficiency as observed in Morogoro WSPs.

Despite the observed decrease in parasite concentrations in the anaerobic ponds, some parasite species showed fluctuations in their mean concentrations as they passed through each WSP treatment stage. In all WSP systems, all parasite species decreased as they passed through the anaerobic ponds, except for *Giardia lamblia* cysts in Morogoro WSPs (Table 2). *Trichostrongylus* spp eggs and *Cryptosporidium* spp oocysts in the maturation ponds of Mwanza and Iringa WSPs, respectively, were found to have higher concentrations in the effluents than the influents (Tables 3 and 4). Most hookworm eggs were cleared in the anaerobic pond in Iringa WSPs, but were observed also in the effluent of the final maturation pond. In the same system, *Taenia* eggs which were not detected in the raw wastewater, or effluent of anaerobic and facultative ponds, were isolated in the effluent of the final maturation pond (Table 4). The main reason for the observed fluctuations in parasite mean concentrations may be due to the reasons explained in Section 3.2 paragraph 3.

Effluents of the final maturation ponds in Mwanza and Iringa WSPs contained both helminth and protozoan parasites. The effluent of the final maturation pond in Morogoro WSPs contained protozoan parasites and no helminth eggs (100% diminution). The mean helminth concentrations in effluents was 2.5 and 7.5 eggs L^−1^, which was equivalent to 96.2% and 85% diminution in Mwanza and Iringa WSPs, respectively. The mean helminth concentrations of effluents in Mwanza and Iringa WSPs corresponded to an average of 5.8 eggs L^−1^ based on data collected by Zacharia et al. [20]. However, studies conducted in Kenya, Tunisia, and Burkina Faso reported complete removal of helminths in WSP systems [32, 35–37]. Except for Morogoro WSPs, the other two systems did not meet the standard set by the World Health Organization (WHO) of ≤1 egg L^−1^ for safe use of wastewater effluents in agriculture [38].

Protozoan concentrations in effluents were 4.3, 0.3, and 1 (oo)cysts L^−1^ in Morogoro, Mwanza, and Iringa WSPs, respectively. These concentrations resulted from 98.8% (2 log units), 99.8% (3 log units), and 99.9% (3 log units) reductions of the initial protozoan concentrations of raw/untreated wastewater in Morogoro, Mwanza, and Iringa WSPs, respectively. According to the WHO guideline, protozoa reduction by wastewater treatment systems of 2–3 log units combined with other health protection measures can achieve the protozoa health-based target for wastewater reuse in restricted, unrestricted, and localized (drip) irrigations [38]. Therefore, the target for protozoa pathogen prevention for people using effluents of the three WSP systems will be achieved if the attained protozoa removal efficiencies of the three treatment systems are sustained and proper health education is given to the exposed communities.

Hookworm eggs, *Trichostrongylus* spp eggs, *Cryptosporidium* spp oocysts, and *Entamoeba coli* cysts were observed to persist throughout all treatment stages of at least one WSP system. Sedimentation is said to be an effective removal mechanism for helminth eggs and protozoan (oo)cysts in WSP systems [39]. The process is greatly influenced by the system's hydraulic retention time (HRT). However, one of the factors that may affect HRT includes sludge accumulation [10]. In addition to that, other design factors may also affect the parasites' residence time distribution in the stabilization ponds, and therefore their sedimentation rate. These factors include pond depth, the length-to-width ratio, the configurations of inlets and outlets, the speed and direction of the wind, stratification caused by diurnal shifts in the temperature at the surface of the pond, and the presence or absence of baffles [39]. Thus, regular system maintenance (disludging) and monitoring is very important to enhance WSP parasite removal efficiency.

### 3.4. Fecal Coliform Bacteria Concentrations and Removal from Wastewater

To monitor all pathogenic organisms that could be present in a particular wastewater treatment system or its effluent is very difficult as it is costly and time-consuming. Indicator organisms are always employed to reflect the pathogen level in a wastewater of a particular treatment system. FCs are bacteria found in the intestines of warm-blooded animals and are used to indicate the presence of human pathogens in wastewater [19]. Compared to other indicators, FC bacteria are the organisms most commonly used to monitor the removal of pathogens from wastewater treatment plants [40].  Table 5 presents concentrations of FCs in influents and effluents of each treatment stage of the three systems. The influent of Iringa WSPs had the highest FC concentration, followed by the Morogoro WSP system. Observed variations in influent FC concentrations were not statistically significant, as indicated by the Kruskal–Wallis test (*H* (2) = 3.01, *p*=0.22). The same test showed that there was significant difference in FC concentrations between effluents of the three WSP systems (*H* (2) = 9.10, *p*=0.01), with a mean rank of 9.60 for Morogoro WSPs, 3.00 for Mwanza WSPs, and 10.50 for Iringa WSPs. The pairwise comparison test indicated that the difference in FC concentrations between effluents of the three WSP systems was significant between Mwanza WSPs and Morogoro WSPs (*p*=0.013), and Mwanza WSPs and Iringa WSPs (*p*=0.008). There was no significant difference between FC concentrations in effluents of Morogoro WSPs and Iringa WSPs (*p*=0.748). The effluent mean FC concentrations from the three systems were higher than that found by Kihila et al. [3] in Moshi WSPs (1.00 × 10^3^ cfu (100 mL)^−1^), and less than that obtained by Kaseva et al. [18] in Lugalo WSPs (8.00 × 10^6^ cfu (100 mL)^−1^).

Generally, Mwanza WSPs achieved more FC removal efficiency (3.81 log units (100 mL)^−1^) than Morogoro WSPs (2.57 log units (100 mL)^−1^) or Iringa WSPs (1.99 log units (100 mL)^−1^). The reason for higher FC removal efficiency in Mwanza WSP systems may be due to the large number of ponds connected in series and increased wastewater temperature. Moreover, Mwanza WSPs have an estimated HRT of 12 days, while Morogoro and Iringa WSPs have the theoretical HRT of 9 days and 5 days, respectively. In WSP systems, the presence of more ponds connected in a series, increase in wastewater temperature, and longer HRT increased reduction of bacteria including FCs [39]. Except for the effluent of Mwanza WSPs, the other systems did not meet the TBS limit for wastewater discharge of 1.00 × 10^4^ cfu (100 mL)^−1^ [12]. According to the WHO guideline, for a wastewater treatment system with FC removal efficiency between 2 and 4 log units or effluent FC (*E*. *coli*) concentration of 1.00 × 10^3^–1.00 × 10^5^ cfu (100 ml)^−1^, its effluent may be applied in various types of agricultural irrigation, as illustrated in Table 6 [38]. In this case, the effluent from Mwanza WSPs can be used for unrestricted or restricted irrigation. However, other health protection measures, such as normal household washing of salads and vegetables with clean and safe water prior to consumption, should be incorporated to achieve the set health-based targets.

### 3.5. Relationship between Physicochemical Parameters and FC Bacteria and Parasites in WSP Wastewater

Physicochemical parameters are among the primary factors involved in the removal and/or destruction of pathogens (parasites and bacteria) in wastewater [41]. To assess the relationship of various physicochemical parameters on the concentrations of FCs, helminths, and protozoa in wastewater as they pass through the three WSPs, a Spearman rho correlation test was conducted. COD, TSS, and TN showed positive correlation with FCs, helminths, and protozoa in the wastewater as they passed through the treatment systems. The correlation was statistically significant between COD and the parasitic organisms (helminths and protozoa). TSS and TN showed statistically significant relationships with helminths only (Table 7). This indicates that physicochemical parameters (COD, TSS, and TN), parasites (helminths and protozoa), and FC bacteria decrease as wastewater passes through WSP systems. Similar relationships were observed by other researchers. Wastewater TSS and nitrogen contents were shown to positively correlate with helminths and protozoa in WSPs in Spain by Reinoso et al. [41]. Moreover, Jimenez and Chavez [42] and Tyagi et al. [43] reported positive correlations between TSS and helminth eggs, and TSS and FCs in WSP wastewater. It has been stated that pathogen particles can effectively be removed by sedimentation if they are attached to larger particles, in which case their elimination from the wastewater correlates with particle removal [44]. That could be the reason for the positive correlation between TSS and indicator bacteria and parasites in WSP wastewater.

Wastewater pH and temperature correlated negatively with FC, helminth, and protozoan concentrations in WSP wastewater. The correlation was statistically significant between pH and parasitic organisms (helminths and protozoa) only (Table 7). In wastewater, a negative correlation between temperature and indicator bacteria was also reported by Liu et al. [19], while temperature and protozoa were reported by Molleda et al. [45]. Effects of temperature and pH on survival and removal of indicator bacteria in WSP have been studied by Liu et al. [19] and Pearson et al. [46], whereby increased temperature and pH were associated with decreased fecal indicator (FC) concentration in WSP systems. The results indicate that protozoan parasites are more affected by an increase in pH and temperature than helminths. An experimental study conducted by Mills [47] in Spain showed that an increase in pH and temperature resulted in an increased disinfection of *Cryptosporidium* spp. According to the author, it is probably as a result of an increase in the permeability of oocyst walls, allowing highly reactive molecules such as hydrogen peroxide and ammonia to penetrate inside the oocysts, increasing their disinfectant effect. The effect could apply to other protozoan species.

### 3.6. Relationship between FCs, Protozoa, and Helminths in WSP Wastewater

The validity of the fecal indicator is determined using the strong correlation of its presence in wastewater and the presence of human fecal contaminants (fecal pathogens). To determine the correlation between FCs and parasites (helminths and protozoa) in wastewater of the three WSPs, a Spearman rho correlation test was conducted (Table 8). The test results indicated a strong positive significant correlation between FC and protozoa concentrations (*r* = 0.604, *p* < 0.01). Similar results were also obtained by Levantesi et al. [48] in Europe, Payment and Locas [49] in Canada, and Reinoso et al. [41] in Spain. Harwood et al. [50] in the United States did not find a correlation between the two parameters. The same test indicated that there was no correlation between FCs and helminths (*r* = 0.051). This result is in accordance with the result reported by Levantesi et al. [48] in Europe, whereby no correlation between the bacteria indicator and helminth eggs was observed. Protozoa and helminths were observed to have moderate positive significant correlations (*r* = 0.30 and *p*=0.04). The positive correlations between protozoa and the other two types of organisms (FCs and helminths) indicate that there are factors affecting the presence of both protozoa and FCs, and factors affecting the presence of protozoa and helminths in WSP wastewater. Both FCs and protozoa in WSPs are affected by factors such as temperature, pH, and sunlight exposure, whereby both FC (bacteria) and protozoa removal increase at increased temperatures, pH, and sunlight exposure [39]. Sedimentation is the main mechanism for pathogen removal in WSPs. The rate of pathogen sedimentation in WSPs varies based on their settling velocity. Protozoan (oo)cysts and helminth eggs have higher settling velocities of 0.026–0.13 m/d and 5–13 m/d, respectively, than bacteria (0.012 m/d) [39]. The higher settling velocities of protozoa and helminths lead to higher removal of these organisms in WSPs than bacteria.

A simple binary logistic regression test was conducted to assess the ability to use FC indicator bacteria to predict the presence of helminth eggs and protozoan (oo)cysts in WSP wastewater. Results indicate that FCs are a good predictor of protozoa occurrence in WSP wastewater (presence/absence) [Chi-square = 6.516, d*f* = 1, and *p*=0.011]. The predictor (FC) explains 15.5% (Nagelkerke *R*^2^) of the variability of protozoa occurrence, which is significant at the 5% level [Wald = 5.708, *p*=0.017 (<0.05)]. The odds ratio is 1.733 (95% CI 1.104–2.720). The model correctly predicted 29.4% of cases where there was an absence of protozoa, and 92.3% of cases where there was the presence of protozoa, giving an overall percentage correct prediction rate of 73.2%. These results are in accordance with results obtained by Payment and Locas [49] and Harwood et al. [50, whereby bacteria indicator/FCs were observed to be a better predictor of protozoa occurrence in wastewater. The same test showed that FCs are not a good predictor of helminth presence or absence in WSP wastewater [Chi-square = 0.360, d*f* = 1, and *p*=0.548].  Table 9 shows the number of correctly and incorrectly predicted samples for protozoa occurrence in wastewater of the three WSP systems.

## 4. Conclusion

The studied WSP reduced pathogenic parasites and FC concentrations from wastewater. However, they did not meet either one or both parasitological and microbiological standards as stated in the WHO guideline and TBS regulations. The Mwanza WSP system achieved the WHO standard for FC bacteria effluent concentration for safe reuse of wastewater in both restricted and unrestricted irrigation. The effluent FC concentration was also in accordance with the TBS standard limit for wastewater discharge into the environment. But the system did not meet the helminth concentration of ≤1 egg/L, as described in the WHO guideline. The Morogoro WSP system meets the helminth standard set by the WHO, but did not meet the WHO and TBS effluent FC concentration standards. The Iringa Municipal WSP system did not achieve either parasitological or microbiological quality as described by both the WHO guideline and TBS regulations. In order to reduce the risk of parasitological and bacterial disease transmission to the exposed community, measures such as proper operation and maintenance of WSPs must be taken. These measures will enhance WSP efficiency and ensure that the parasitological and microbial quality of effluents is achieved and sustained. Some efforts were observed in the Iringa Municipal WSP, whereby two new maturation ponds were under construction during the study period. Perhaps the addition of these two ponds will improve the parasitological and microbiological quality of the effluent of this system to a level that adheres to the WHO and TBS requirements.

FCs correlated with protozoan (oo)cysts, but not with helminth eggs. Binary logistic regression results indicated that FCs are a good predictor of protozoa occurrence in wastewater but not of helminths. Thus, FCs are not a reliable indicator of helminths in wastewater. Therefore, during wastewater monitoring for reuse or discharge into sensitive receiving water bodies, such as lakes, rivers, and streams, helminth quality must be surveyed independently.

## Figures and Tables

**Table 1:** Physicochemical characteristics of influents and effluents of wastewater of the three WSP systems.

Parameter	Morogoro WSPs			Mwanza WSPs			Iringa WSPs		
	Inf	Eff	% removal	Inf	Eff	% removal	Inf	Eff	% removal
COD (mg/L)	420	200	52.4	575	215	63	815	235	71.2
TSS (mg/L)	135	57	58	311	105	66	494	154	69
TN (mg/L)	39	32	19	57	38	33	65	52	19
pH	7.5	8.3		7.4	8.2		7.0	8.1	
Temperature (°C)	29	29		28	30		23	28	

*Note*. COD = chemical oxygen demand; TSS = total suspended solids; TN = total nitrogen; Inf = influents; Eff = effluents.

**Table 2:** Parasite concentrations and removal in the Morogoro WSP system.

Parasite group	Parasite species	Treatment stage			
		Raw wastewater	Anaerobic pond	Facultative pond	Maturation ponds
Helminth species concentrations (egg/L)	*Ascaris lumbricoides*	12	0.8	0	0
	Hookworm	0	0	0	0
	*Trichuris trichiura*	0	0	0	0
	*Trichostrongylus* spp	0	0	0	0
	*Fasciola* spp	0	0	0	0
	*Taenia* spp	0	0	0	0
Total helminth concentration (egg/L)		12	0.8	0	0
Helminth removal at each stage (%)			93.3	100	—
Helminth removal from raw wastewater (%)			93.3	100	100
Protozoa species concentrations ((oo)cysts/L)	*Giardia lamblia*	22	32.4	5.4	0
	*Entamoeba histolytica/dispar*	26	15.6	0.3	0
	*Entamoeba coli*	248	194.8	47.9	0.8
	*Cryptosporidium* spp	72	60.4	23.7	3.5
	*Isospora* spp	0	0	0	0
Total protozoa concentration ((oo)cysts/L)		368	303.2	77.3	4.3
Protozoa removal at each stage (%)			17.6	74.5	94.4
Protozoa removal from raw wastewater (%)			17.6	79	98.8

**Table 3:** Parasite concentrations and removal in the Mwanza WSP system.

Parasite group	Parasite species	Treatment stage			
		Raw wastewater	Anaerobic ponds	Facultative ponds	Maturation ponds
Helminth species concentration (eggs/L)	*Ascaris lumbricoides*	19	0	0	0
	Hookworm	32	1.8	0.8	0.2
	*Trichuris trichiura*	0	0	0	0
	*Trichostrongylus* spp	8	0.4	12.2	2.3
	*Fasciola* spp	8	0	0	0
	*Taenia* spp	0	0	0	0
Total helminth concentration (eggs/L)		67	2.2	13	2.5
Helminth removal at each stage (%)			96.7	No reduction	80.8
Helminth removal from raw wastewater (%)			96.7	80.6	96.2
Protozoa species concentrations ((oo)cysts/L)	*Giardia lamblia*	9	1.6	0.2	0
	*Entamoeba histolytica/dispar*	80	3.2	0	0
	*Entamoeba coli*	127	5.6	1	0
	*Cryptosporidium* spp	12	1.6	0.6	0.3
	*Isospora* spp	3	0	0	0
Total protozoa concentration ((oo)cysts/L)		231	12	1.8	0.3
Protozoa removal at each stage (%)			94.8	85	83.3
Protozoa removal from raw wastewater (%)			94.8	99.2	99.8

**Table 4:** Parasite concentrations and removal in the Iringa WSP system.

Parasite group	Parasite species	Treatment stage			
		Raw wastewater	Anaerobic ponds	Facultative pond	Maturation ponds
Helminth species concentrations (eggs/L)	*Ascaris lumbricoides*	10	3	0	0
	Hookworm	9.5	0	0	7.5
	*Trichuris trichiura*	20	4.5	0	0
	*Trichostrongylus* spp	10	4.5	0	0
	*Fasciola* spp	0	0	0	0
	*Taenia* spp	0	0	0	0.25
Total helminth concentration (eggs/L)		49.5	12	0	7.75
Helminth removal at each stage (%)			75.8	100	No reduction
Helminth removal from raw wastewater (%)			75.8	100	85
Protozoa species concentrations ((oo)cysts/L)	*Giardia lamblia*	0	0	0	0
	*Entamoeba coli*	582.5	49.3	7.5	0.75
	*Entamoeba histolytica/dispar*	72	6	0	0
	*Cryptosporidium* spp	15	0	2	0.25
	*Isospora* spp	0	0	0	0
Total protozoa concentrations ((oo)cysts/L)		669.5	55.3	9.5	1
Protozoa removal at each stage (%)			91.7	82.8	89.5
Protozoa removal from raw wastewater (%)			91.7	98.6	99.9

**Table 5:** FC concentration means of raw wastewater and effluents of anaerobic, facultative, and maturation ponds of the three WSP treatment systems.

Type of wastewater	Morogoro WSP		Mwanza WSP		Iringa WSP	
	Cfu/100 ml	Log units/100 ml	Cfu/100 ml	Log units/100 ml	Cfu/100 ml	Log units/100 ml
Raw wastewater	2.07 × 10^8^	7.95	1.01 × 10^8^	7.43	3.12 × 10^8^	8.06
Anaerobic pond(s)	1.14 × 10^8^	7.53	4.42 × 10^6^	6.52	8.15 × 10^7^	7.13
Facultative pond(s)	3.21 × 10^6^	6.39	2.73 × 10^5^	5.21	2.61 × 10^7^	6.56
Maturation pond(s)	5.81 × 10^5^	5.38	4.92 × 10^3^	3.62	5.73 × 10^6^	6.07

*Note*. WSP = waste stabilization pond; cfu = colony-forming unit.

**Table 6:** Bacterial quality requirement for wastewater effluent reuse in agricultural irrigation.

Type of irrigation	WTS required FC (*E*. *coli*) reduction (log units)	Effluent FC (*E*. *coli*) concentration during monitoring (cfu/100 ml)	Notes
Unrestricted	4	≤10^3^	Root crops
	3	≤10^4^	Leaf crops
	2	≤10^5^	Drip irrigation of high-growing crops

Restricted	4	≤10^3^	Drip irrigation of low-growing crops
	3	≤10^4^	Labour-intensive agriculture
	2	≤10^5^	Highly mechanized agriculture

*Note*. Table modified from the WHO 2006 guideline. WTS = wastewater treatment system.

**Table 7:** Spearman's rho correlation test results for physicochemical parameters and microorganisms (including helminths).

Parameter	Helminths (eggs/L)			Protozoa ((oo)cysts/L)		FCs (cfu/100 ml)	
	*n*	*r*	*p*	*r*	*p*	*r*	*p*
COD (mg/L)	12	0.71	0.009	0.78	0.003	0.53	0.079
TSS (mg/L)	12	0.59	0.041	0.48	0.118	0.48	0.137
TN (mg/L)	12	0.74	0.005	0.26	0.421	0.26	0.420
pH	12	−0.594	0.042	−0.71	0.010	−0.57	0.055
Temperature (°C)	12	-0.367	0.241	−0.57	0.054	−0.50	0.094

*Note*. COD = chemical oxygen demand; TSS = total suspended solids; TN = total nitrogen; *n* = number of datasets; *r* = Spearman's correlation coefficient. *p* is significance level.

**Table 8:** Spearman's rho correlation test results for FCs, helminths, and protozoa.

Microorganism	Number of samples	Mean	Median	FCs		Protozoa		Helminths	
				*r*	*p*	*r*	*p*	*r*	*p*
FCs (cfu/100 ml)	56	6.8 × 10^7^	4.8 × 10^6^	—	—	sc	s	nc	n
Protozoa ((oo)cysts/L)	56	141.6	6.0	0.60	0.000	—	—	Mc	s
Helminths (eggs/L)	56	13.7	0	0.05	0.709	0.30	0.049	—	—

*Note*. cfu = colony-forming unit; *r* = Spearman's correlation coefficient; *p* = is significant level; sc = strong correlation; s = significant; nc = no correlation; n = not significant; wc = moderate correlation.

**Table 9:** Protozoa prediction results by FC indicator bacteria in WSP wastewater.

Observed		Predicted		
		Protozoa occurrence		
		Negative	Positive	Percentage correct
Protozoa occurrence	Negative	5	12	29.4
	Positive	3	36	92.3
Overall percentage			73.2	

The cut-off value is 0.500.

## Data Availability

The data used to support the findings of this study are available from the corresponding author upon request.
